# Non-therapeutic plasma levels in individuals utilizing curcumin supplements in daily life

**DOI:** 10.3389/fnut.2023.1267035

**Published:** 2023-11-30

**Authors:** Maurice A. G. M. Kroon, Jacqueline K. Berbee, Soumia Majait, Eleonora L. Swart, Olaf van Tellingen, Hanneke W. M. van Laarhoven, E. Marleen Kemper

**Affiliations:** ^1^Department of Pharmacy and Pharmacology, Amsterdam UMC location AMC, Amsterdam, Netherlands; ^2^Department of Pharmacy and Pharmacology, The Netherlands Cancer Institute-Antoni van Leeuwenhoek Hospital, Amsterdam, Netherlands; ^3^Department of Medical Oncology, Cancer Center Amsterdam, Amsterdam UMC, University of Amsterdam, Amsterdam, Netherlands; ^4^Department of Experimental Vascular Medicine, Amsterdam UMC location AMC, Amsterdam, Netherlands

**Keywords:** curcumin, curcuminoids, tetrahydrocurcumin, piperine, β-glucuronidase, HPLC-MS/MS

## Abstract

**Introduction:**

The spice curcumin and its metabolites are widely used by cancer patients but have not shown proven health benefits in clinical studies, likely due to low plasma concentrations after oral intake. However, public interest in curcumin continues to grow, and companies claim enhanced absorption in their formulations. This study aims to determine if daily oral intake of curcumin leads to sufficient plasma concentrations for health effects. The study was registered in the Dutch Clinical Trial Register with ID NL5931.

**Methods:**

We used a validated HPLC-MS/MS method to measure curcumin and its metabolites in 47 individuals using their own curcumin formulations. Questionnaires assessed other supplement and medication use. Plasma samples were collected before and 1.5 h after intake, analyzing curcumin and metabolite levels with and without β-glucuronidase pretreatment to measure conjugated and unconjugated forms.

**Results:**

Plasma concentrations of curcumin, demethoxycurcumin, bisdemethoxycurcumin and tetrahydrocurcumin, ranged between 1.0 and 18.6 ng/mL. Adding β-glucuronidase resulted in an increase of unconjugated curcumin plasma levels to 25.4 ng/mL; however still significantly below (1000-fold) a plasma concentration that is expected to have a beneficial health effect. The use of adjuvants like piperine did not result in higher curcumin plasma concentrations.

**Discussion:**

Our study shows that using oral curcumin supplements still does not result in therapeutic plasma levels. Health care practitioners need to be critical toward the claimed beneficial systemic health effects of current curcumin supplement use by their patients.

**Clinical Trial Registration:**

https://onderzoekmetmensen.nl/en/trial/25480, NL5931.

## 1 Introduction

The spice curcumin originates from the root of the *Curcuma Longa* plant and has been used for centuries in Asian cultures as dietary pigment, spice and traditional medicine. Curcumin has been associated with many beneficial health outcomes, such as anti-inflammatory and anti-cancer effects (1–3). However, these effects have mostly been assessed in *in vitro* experiments. For example, curcumin showed activity against a human pancreatic carcinoma cell-line with IC90 values of 6.75–94.5 μM (4) and reduced the tumor growth in a mouse model for pancreatic cancer (5). Clinical trials, however, could not confirm these results (6, 7). One publication described some beneficial clinical effect in two patients, but it is questionable if this effect can be attributed solely to the use of curcumin (8).

One of the major known issues potentially explaining the low therapeutic effectiveness of curcumin in patients is the low bioavailability after oral administration. Curcumin dosages of up to 12 gram show no detectable levels of curcumin in the systemic circulation (9). The low solubility, low dissolution rate, and instability in intestinal pH of curcumin do not favor absorption from the small intestine (10). Furthermore, curcumin is subjected to extensive phase II metabolism in the gut wall, resulting in low plasma levels of the unconjugated, active curcumin (11, 12). Levels of inactive glucuronidated and sulfated forms of curcumin have been quantified in human plasma, suggesting that active curcumin is converted into inactive metabolites by urididne-5′-diphosphoglucuronic acid (UGTs) in the gut wall.

To overcome the low bioavailability, different curcumin formulations, such as lipid formulations, have been developed. Several of these products are currently highly advertised and used by patients (11). This increase in the use of curcumin-loaded liposomes, micelles, colloidal suspensions, and lipid-based nanoparticles resulted in multiple clinical trials on curcumin nano-formulations registered on clinicaltrials.gov. Furthermore, it has been described that the addition of certain adjuvants could overcome the low bioavailability of curcumin. One clinical study showed that addition of 20 mg/kg piperine increased bioavailability of curcumin by 154% in rats and 2000% in humans (12). Piperine is suggested to inhibit phase I and II metabolism by inhibiting CYP3A4 and UGT, thereby inhibiting the formation of the more watersoluble and excretable metabolites of curcumin (13–15).

Despite the disappointing results in clinical studies and the knowledge of the low bioavailability, the public’s interest in curcumin continues to grow due to new formulations being developed by various manufactures. This is partly due to the supplement manufacturers using β-glucuronidase to hydrolyze the hydrophilic glucuronic and sulfate acid group from conjugated curcumin and thus measuring the sum of conjugated (inactive) and unconjugated (active) curcumin resulting in claims of a high absorption of curcumin in plasma (16–18). If we can assess the plasma concentration of curcumin formulations being used by patients and healthy volunteers in daily life and differentiate between conjugated and unconjugated curcumin and its metabolites, we can better inform patients and healthy individuals about the validity of the supposed claimed beneficial effects of curcumin supplement use. In this independent, observational study, we therefore addressed the following questions: (1) What is the plasma concentration of conjugated and unconjugated curcumin, demethoxycurcumin, bisdemethoxycurcumin, and tetrahydrocurcumin when curcumin is used in daily life? (2) Which adjuvants, such as piperine, do participants use to increase the bioavailability of curcumin, and are they effective?

## 2 Materials and methods

### 2.1 Study participants

All participants were recruited through free advertisements placed inside the Amsterdam UMC hospital, location Academic Medical Centre from 1st of December 2016 till 21st September 2017. Forty-seven adult persons (patients or healthy volunteers) signed the informed consent. The inclusion criteria comprised of age 18 years or older, mentally competent to give informed consent and use of curcumin supplement in daily life. Participants were excluded if they were not mentally competent to sign the informed consent or were incapable of following the instructions of the researcher. Participants were asked to give information about their health condition and provided, where possible, an overview of their prescribed drugs. Un-prescribed medication, use of food supplements and/or other herbal drugs were also recorded. Participants were allowed to participate twice – after a minimal 3-day washout period – with different curcumin formulations. The study protocol was reviewed and approved by the Ethics committee of the Amsterdam UMC, location Academic Medical Centre, The Netherlands (approval 2016_201#B2016569). The study was registered in the Dutch Clinical Trial Register with ID NL5931.

### 2.2 Study design

This study is an observational, open-label study investigating the plasma concentration of curcumin as used in daily life. Each participant was instructed to bring their own curcumin formulation on the day of visit. Participants were asked not to take their curcumin formulation in the morning on the day of visit. Blood was taken just before intake of the curcumin supplement (trough concentration) and 1.5 h after intake (expected peak concentration). Any food or beverage intake between these time points was registered while participants were not allowed to take any additional curcumin in between these two time points.

### 2.3 Blood sampling and processing

Blood was collected in heparinized tubes and protected against light by wrapping in aluminum foil directly after sampling. All samples were centrifuged (Rotina 380R from Hettich) for 10 min at 2754 RCF and 20°C and the obtained plasma was immediately stored, protected from light, at –80°C until analysis.

### 2.4 Addition of the enzyme β-glucoronidase

**Figure 1:**
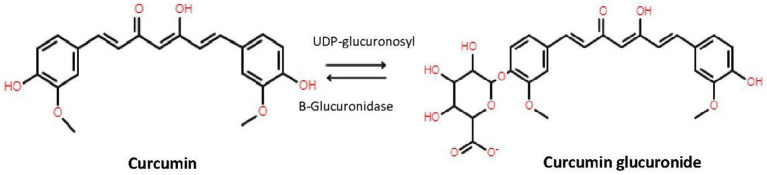
Conversion of curcumin by UDP-glucuronidase to curcumin and back to curcumin to β-glucuronidase.

β-glucuronidase is an endogenous enzyme and is distributed in mammalian tissues, body fluids and microbiota mainly as a lysosomal hydrolase; but significantly retained in the endoplasmic reticulum (19). To determine the active plasma concentrations of curcumin, demethoxycurcumin, bisdemethoxycurcumin, and tetrahydrocurcumin it is important to highlight the difference between conjugated and unconjugated forms of these different compounds. A way to show this difference is to pretreat each sample with and without β-glucoronidase. In short, we used β-glucoronidase from Sigma-Aldrich (St. Louis, MO, USA), which contained ≥300,000 U/g β-glucoronidase and ≥10,000 U/g sulfatase, which hydrolyzes the hydrophilic glucuronic and sulfate acid group from conjugated curcumin (Figure 1) (19). The optimal pH for β-glucoronidase activity is 5.0 (20). Although our final mixture of sample plus β-glucoronidase solution has a slightly higher pH, we confirmed that after 1 h incubation at 37°C and 850 repetition per minute (rpm), a plateau was reached. No further conversion of conjugated curcumin into deconjugated curcumin occurred, indicating a complete reaction. Therefore, adding β-glucoronidase/sulfatase mixture results in an increase of unconjugated curcumin, demethoxycurcumin, bisdemethoxycurcumin, and tetrahydrocurcumin by displaying the sum of ‘previous’ conjugated and unconjugated curcuminoids and tetrahydrocurcumin.

### 2.5 Analysis of curcumin, demethoxycurcumin, bisdemethoxycurcumin, tetrahydrocurcumin and piperine in plasma

Plasma samples were analyzed by our validated HPLC-MS/MS method (21). In short, on the day of analysis, samples were thawed and pretreated with and without β-glucoronidase. The samples treated with β-glucoronidase were incubated with 20 U/μL β-glucuronidase at 37°C and 850 revolutions per minute (RPM) for 1 h. Liquid–liquid extraction with Tert Butyl Methyl Ether (TBME) as organic phase was used to extract the compounds from the plasma. Curcumin, demethoxycurcumin, bisdemethoxycurcumin, tetrahydrocurcumin and piperine were quantified in a single analytical run. The HPLC-MS/MS system consisted of an Ultimate 3000 autosampler and pump, both of Dionex, connected to a degasser from LC Packings. The autosampler, with a 100 μL sample loop, was coupled to a Sciex API4000 mass spectrometer. After injection of 50 μL sample, separation of the analytes was performed with an Agilent column 2.1 × 100 mm packed with material of Zorbax Extend 3.5 μm C-18. The flowrate was 0.200 μL/min and the dual gradient mobile phase consisted of A: ultra-purified H2O with 0.1% formic acid and B: MeOH 100%. The applied gradient profile started at 50:50 A:B and increased linear to 95% B in 3.0 min. During 6 min a 5:95 A:B level is continued, after which it returned in 0.2 min to 50:50. Afterwards the system was equilibrated during 6 min at the starting level. Throughout the liquid–liquid extraction and HPLC-MS/MS the potential influence of light was minimized by working in an dark environment with aluminum foil as much as possible. Each analytical run included a set of freshly prepared calibration samples containing all compounds in the validated range of 2 nM to 400 nM.

### 2.6 Statistical analysis and sample size calculation

This is an observational study and the main aim is to assess if curcumin is detectable in the plasma of participants after oral intake. Due to the descriptive nature of this study design with different dosages and different formulations, no statistical analysis was performed. Any curcumin plasma level below the LLQ of 2 nM is not expected to have any health effect since the *in vitro* results were seen at concentrations of 2–100 μM.

Because this study is descriptive in nature and lacks statistical analysis, it is not feasible to calculate the sample size using the standard single-group repeated measures analysis of variance with an alpha level of 0.05 (without adjustment for multiple comparisons). This approach would result in 98% power to detect a difference in the means of the efficacy parameter (plasma concentration of curcumin), characterized by a specific effect size (e.g., a Variance of means of 2.00, a standard deviation of 1.5 at each level, and a between level correlation of 0.0). Therefore, with the widespread usage of curcumin amongst various patient populations and the known low bioavailability (effect) of curcumin, our aim was to include 50 participants. This standard sample size provides preliminary evidence of the plasma concentration curcumin levels reached when curcumin is taken in daily life and gives an adequate indication of the different curcumin products used in daily life.

## 3 Results

### 3.1 Participants and overview of curcumin use

**Figure 2:**
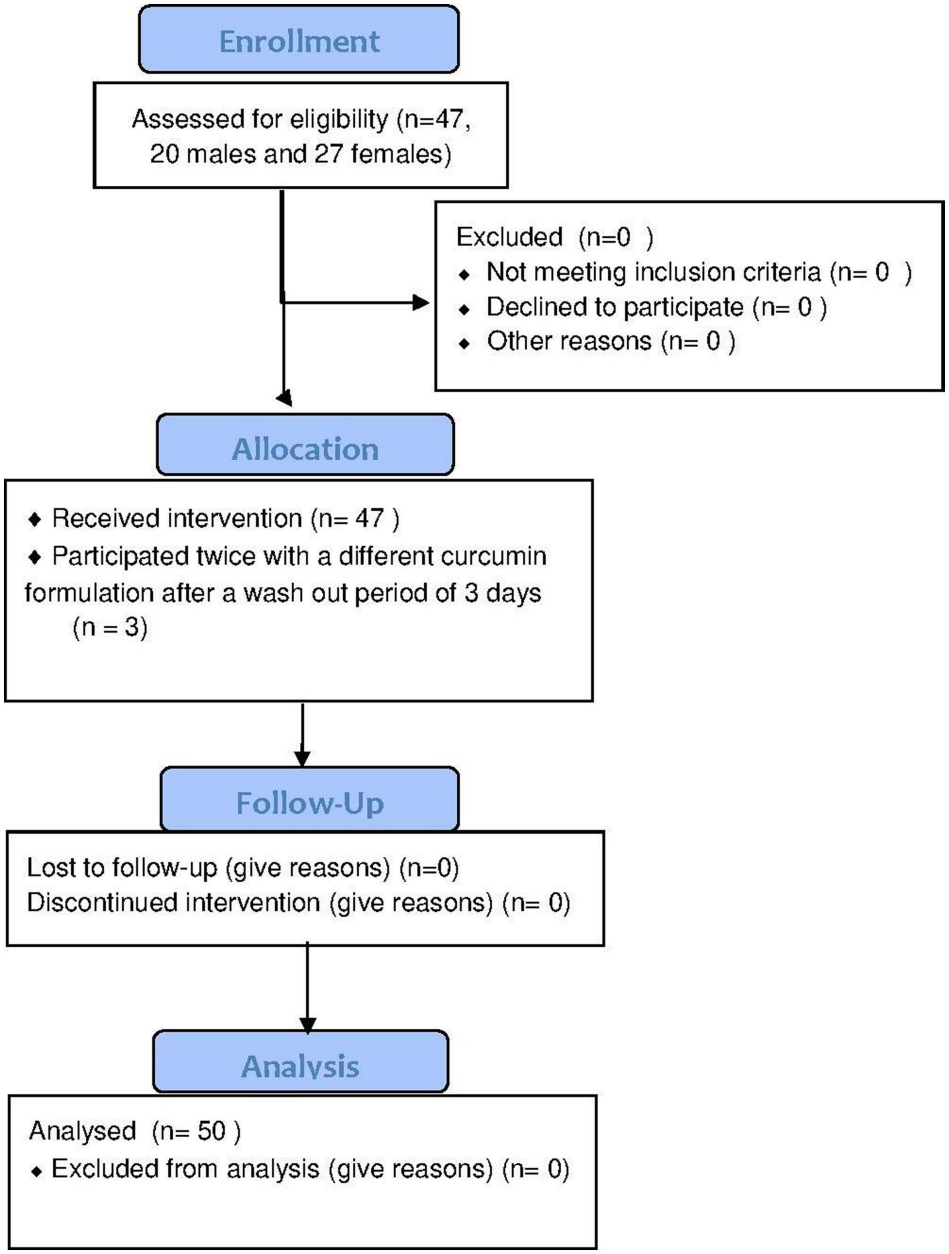
CONSERT Flowchart of enrollment, allocation, follow up and analysis. Forty-seven participants (20 males and 27 females) were included of which 3 participants participated twice resulting in 50 data points.

Within 10 months (December 2016 till September 2017), 47 adult persons were included, 20 males and 27 females (see Figure 2). Three persons participated twice using different curcumin formulations and the interval between their participation was 3 days, 13 days and 31 days, respectively. Among the 47 participants, 170 different food supplements and 95 different prescribed drugs were reported. The food supplements can be divided into seven different categories: vitamins and minerals, herbs and other botanicals, enzymes and antioxidants, fish oil and derivatives, cannabis oil, sedatives and miscellaneous (Table 1). Vitamins and minerals were most frequently reported, followed by herbs and other botanicals. We assessed which herbal products or supplements were used that might influence the bioavailability of curcumin. Piperine (used by most participants as additive in their curcumin supplement), genistein or drugs metabolized by the same enzymes as curcumin (CYP3A4, CYP2C9, UGT and SULT) have a potential effect on influence the bioavailability of curcumin (22). Table 2 describes prescribed drugs used by participants and are divided into different drug classes. Antihypertensive drugs were the most prescribed drug class, followed by corticosteroids.

**Table 1:** Overview of subclasses of food supplement category amongst the 47 participants.

Food supplement category	Frequency
Vitamins and minerals	26
Herbs and other botanicals	16
Enzymes and antioxidants	4
Sedatives and miscellaneous	3
Fish oil and derivatives	2
Cannabis oil	2

**Table 2:** Overview of subclasses of prescribed drugs amongst the 47 participants.

Drug classes	Frequency
Antihypertension/betablockers	13
Corticosteroids	7
Proton pump inhibitors	5
Vitamins	5
Thyroid drugs	4
Anticoagulants	4
Cholesterol lowering drugs	3
Painkillers	3
Antidiabetic	3
Hormones	3
Renal	3
Allergy	3
Anti-emeticum	3
Anti-diuretic	2
Antibodies	2
OTC	2
Psychopharmaca (SSRI/benzodiazepine)	3
Birth control	1
Anti-epileptic	1
Cytostatic	1
Enzymes	1
Antiviral	1
Laxatives	1
Total	74

**Figure 3:**
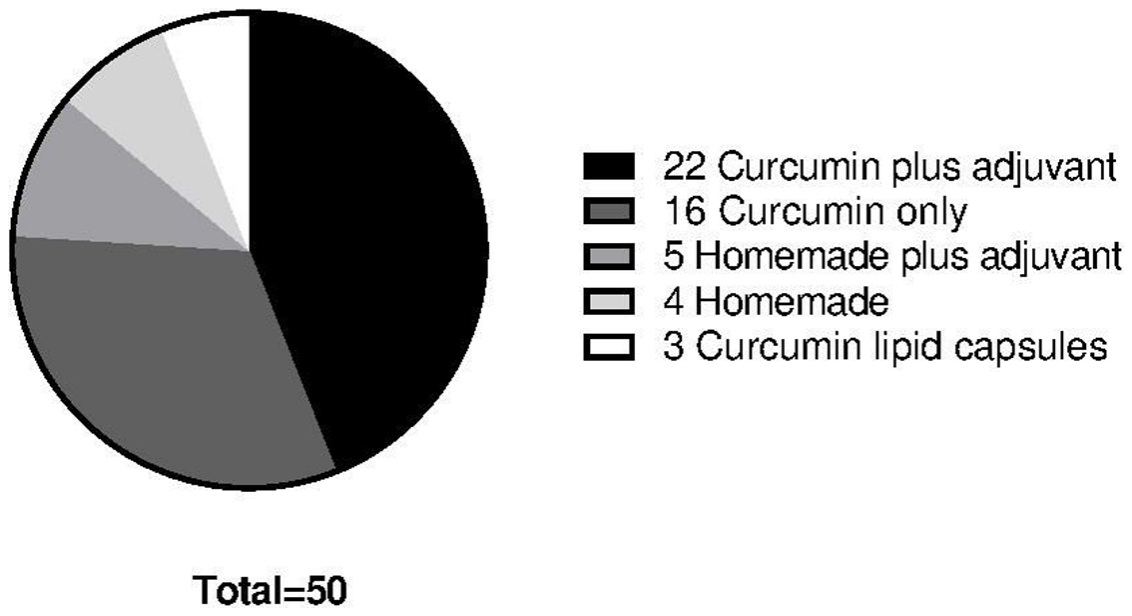
Different formulations of curcumin used among the 47 participants totaling in 50 data points. Twenty-two participants used curcumin plus an adjuvant of which 22 formulations consisted of different brands curcumin capsules with added piperine and different dosages. Sixteen participants used curcumin only supplements of which 16 formulations consisted of different curcumin capsule brands in different dosage. Five participants used homemade formulations plus an adjuvant of which everybody used piperine as adjuvant. Four participants used a homemade formulation consisting of crude curcumin powder. Three participants used curcumin only in a lipid capsule formulation.

The 47 participants used in total 34 different curcumin formulations with dosages ranging from 200 mg to 3000 mg (~140 mg to ~2100 mg of curcumin). Sixteen participants used commercially available curcumin capsules without adjuvants, whereas 22 participants used curcumin in combination with an adjuvant (mostly piperine). Three participants used a curcumin formulation where curcumin micelles were formulated in soft gel capsule. Four participants made their own curcumin formulation (i.e., tea or in food). Five participants made their own formulations with adding adjuvants like piperine (Figure 3).

### 3.2 Plasma levels of conjugated and unconjugated curcumin, demethoxycurcumin, bisdemethoxycurcumin and tetrahydrocurcumin and the adjuvant piperine

All plasma samples were analyzed with and without the addition of β-glucuronidase to determine unconjugated and conjugated curcumin. The analysis of curcumin, demethoxycurcumin, bisdemethoxycurcumin, tetrahydrocurcumin and piperine of the 47 participants are shown in Table 3. Without the addition of β-glucuronidase, curcumin was only detected at the trough level in one of the participants at 10.1 ng/mL. At the expected peak levels, 1.5 h after intake, curcumin was detected in 2 participants at 0.9 and 1.1 ng/mL. Demethoxycurcumin plasma levels were below the lowest limit of quantification (LLQ) of 0.68 ng/mL at through and expected peak level. Bisdemethoxycurcumin was detected in one participant at 2.1 ng/mL at the trough level whereas at the expected peak level the concentration remained below the LLQ of 0.62 ng/mL in all participants. Tetrahydrocurcumin was below the LLQ of 0.74 ng/mL at trough levels and only detected at the expected peak levels in two participants with concentrations of 16.4 and 20.9 ng/mL.

**Table 3:** Results of curcumin, demethoxycurcumin, bisdemethoxycurcumin, tetrahydrocurcumin and piperine in their conjugated and unconjugated forms in human plasma at through and expected peak level.

		Without addition of β-glucuronidase		with addition of β-glucuronidase	
Compound (LLQ in ng/mL)	Time of intake (hour)	Detectable levels in # persons	Mean + Concentration range (ng/mL)	Detectable levels in # persons	Mean + Concentration range (ng/mL)
Curcumin (0.74 ng/mL)	0	1	10.1	27	14.3 (0.8–66.3)
	1.5	2	1.0 (0.9–1.1)	42	25.4 (0.7–130.8)
Demethoxycurcumin (0.68 ng/mL)	0	0	*n.d*.	9	3.5 (1.4–8.6)
	1.5	0	*n.d.*	26	9.4 (0.9–73.1)
Bisdemethoxycurcumin (0.62 ng/mL)	0	1	2.1	8	1.6 (0.7–2.7)
	1.5	0	*n.d.*	25	6.1 (0.7–60.4)
Tetrahydrocurcumin (0.74 ng/mL)	0	0	*n.d.*	30	14.6 (0.8–81.2)
	1.5	2	18.6 (16.4–20.9)	39	21.6 (1.0–161.6)

All compounds are shown before and after the addition of β-glucuronidase. Addition of β-glucuronidase leads to an increase in unconjugated forms of curcumin. *n.d*. = not detectable (below the LLQ).

After treatment of the samples with β-glucuronidase, 27 participants showed unconjugated curcumin levels at trough levels varying from 0.78 to 66.3 ng/mL and 42 participants showed unconjugated curcumin levels at 1.5 h after dosing (expected peak level) varying from 0.7 to 130.8 ng/mL. The concentration of curcumin after hydrolysis could not be detected (below our LLOQ) in 5 participants. Of these 5 participants, 4 participants also did not show any curcumin level above our LLOQ at the expected trough concentration. Of these 4 participants, 3 participants used a commercially available curcumin formulation with curcumin dosages of 200 mg, 400 and 600 mg. One of these 4 participants used a homemade recipe. One of the 5 participants did show a curcumin concentration (just above our LLOQ) at trough concentration and was using commercial product with a dosage of 600 mg curcumin. The three participants using curcumin lipid formulations showed unconjugated plasma concentrations of 80.7–130.8 ng/mL. Demethoxycurcumin peak level concentrations were detected in 26 persons with varying concentrations of 0.9–73.1 ng/mL. Bisdemethoxycurcumin peak level concentrations were detected in 25 persons with concentrations varying from 0.7 to 60.4 ng/mL. Tetrahydrocurcumin peak level concentrations were detected in 39 persons with concentrations varying from 1.0 to 161.6 ng/mL. Piperine trough and peak level concentrations were also assessed and ranged from 1.0 to 1096 ng/mL and were detectable in all participants whether or not it was purposely used as adjuvant to curcumin.

## 4 Discussion

Our study shows that there is very little to no systemic exposure of curcumin, demethoxycurcumin, bisdemethoxycurcumin and tetrahydrocurcumin, when used in daily life. Adjuvants or use of different formulations did not result in therapeutic concentrations of curcumin in plasma. Our study underscores the need to be critical to the claimed beneficial effects of curcumin supplement use in daily life among patients and healthy subjects (22).

Our first aim was to assess the plasma concentration of curcumin, demethoxycurcumin, bisdemethoxycurcumin, tetrahydrocurcumin, and the adjuvant piperine. The *in vitro* results described in the literature show effective curcumin concentrations between 2 and 100 μg/mL (4, 23–26). However, our results show that unconjugated, free curcumin (the active form) does not reach concentrations above 0.74 ng/mL in the plasma of our participants, which is more than a 1000-fold lower than the concentrations assessed *in vitro*. This finding implicate that the achieved systemic curcumin concentrations at everyday use is too low to expect systemic health effects.

Our results confirm the several studies claiming absorption of curcumin and thus high systemic concentrations of curcumin (27, 28). However, we distinguish between conjugated and unconjugated curcumin and other studies only report total curcumin (including inactive metabolites). In Table 3 we showed that without the addition of β-glucuronidase the curcumin levels were undetectable. However, when β-glucuronidase was added, curcumin levels increased to detectable levels. As mentioned in section 2.4, the β-glucuronidase enzyme hydrolyzes the hydrophilic glucuronic group where sulfatase hydrolyzes the sulfate acid group from curcumin (Figure 1), thus increasing the levels of unconjugated curcumin and its metabolites (29). Our findings showed detectable plasma levels of curcumin in 42 participants after the addition of β-glucuronidase, while curcumin plasma levels assessed without β-glucuronidase were only found in two participants. This suggests that curcumin is conjugated immediately after absorption, indicating a virtually complete first pass metabolism. It is assumed that unconjugated curcumin is responsible for the therapeutic effect, while conjugation reduces the activity (30). Several studies add β-glucuronidase to plasma prior to analysis of blood samples (16–18). These studies claim a higher plasma concentration of up to 185x the concentration of unconjugated curcumin.

The indication that curcumin undergoes rapid and complete phase II metabolism, combined with the poor absorption, prevents the attainment of therapeutic concentrations of curcumin. This was observed in a preclinical study, which showed that after oral administration of 1.5 mg/kg curcumin formulated in a colloidal suspension (Theracurmin™) in Sprague–Dawley rats, the levels of unconjugated curcumin in the portal vein and abdominal portion of the vena cava ranged from 5.1 ± 2.3 to 2.1 ± 0.9 ng/mL, respectively, after 0.5 h. However, the levels of conjugated curcumin in the portal vein and abdominal portion of the vena cava were 87.9 ± 45.2 and 65.1 ± 46.3 ng/mL, indicating a high first-pass metabolism of curcumin (31). In studies with oral lipid formulations where β-glucuronidase was added prior to analysis, curcumin concentrations up to 1189.1 ± 518.8 ng/mL were found (28, 32, 33). Our study demonstrated that only one of the three participants using similar curcumin lipid formulations at a dosage of 800 mg was able to reach a curcumin Cmax of 355 nmol/L. The clinical trial database of privately and publicly funded clinical studies conducted around the world, clinicaltrials.gov, currently report that liposomal curcumin is being tested in clinical trials (NCT01403545, NCT04315350 which stopped inclusion due to COVID). However, to date, no results are reported.

Intravenous formulations could overcome the problems related to the low bioavailability. However, these formulations will be challenging due to the poor stability of curcumin in several solvents (34). At neutral pH and a temperature of 37°C, the chemical half-life (*t*1/2) of curcumin is between 10 and 20 min. The chemical t1/2 decreases by 50% in human blood incubated for 8 h at 37°C (34). Therefore, due to these chemical instability, the availability of curcumin becomes negligible. However, one dose-finding clinical trial in patients with solid tumor cancers (NCT02138955) with intravenous curcumin did publish results. In 23 of the 33 participants, treatment was stopped after cancer disease progression was found. In six patients, the general medical condition deteriorated, with two early withdrawal and one study interruption. This study demonstrates that intravenous administration of liposomal curcumin is far from achieving any beneficial health results.

Our second aim was to determine which adjuvants, such as piperine, or supplements are used by individuals with the aim to increase the bioavailability of curcumin. It is suggested that piperine affects curcumin plasma levels with an IC50 value of 5.5 ± 0.7 μM on CYP3A, and IC50 values between 29.8 and 50 μM on CYP2C9, CYP2D6, CYP2C19, CYP1A2, CYP2E1, and CYP2B6 (35). Twenty-five out of the 47 participants used piperine as adjuvant; however, nearly all participants showed traceable plasma levels of piperine. These traceable plasma levels of piperine were, however, nearly 10-to 60-fold lower than piperine plasma concentrations described in literature that show any substantial effect on cur-cumin bioavailability. Moreover, the results of our study could not confirm the results of the study by Shoba et al. where the bioavailability of curcumin increased by 2000% with addition of piperine (12). In this study 20 mg piperine was added to 2000 mg curcumin, which is comparable to the amounts of the supplements our participants have taken. Other herbal drugs or supplements were unlikely to have an effect on achieving therapeutically levels of curcumin.

Due to the nature of our study design, we are able to establish that little to no systemic effect can be expected with these curcumin plasma levels. However, we cannot rule out any local effects in the lumen of the intestine that curcumin might induce. There are increasing reports of research in animals and humans where curcumin has been described as having a potential beneficial effect on Inflammatory Bowel Diseases (IBD) like Crohn’s disease and Ulcerative Colitis (36). It would, therefore, be more probable to expect beneficial health effects in local gut diseases than to expect a systemic health effect. Future research should assess this.

A limitation of our study is that by allowing the participants to bring their own curcumin formulation, we introduced many variables, such as different dosages and a range of different formulations. Despite the fact that we let the participants take the curcumin in a controlled setting, we cannot guarantee that the curcumin formulations were of good quality. However, the goal of this study was to assess curcumin plasma concentrations in everyday life use, as the participants believe in the benefits of their curcumin use. Future research needs to be conducted to analyze curcumin in a randomized controlled clinical trial where different dosages and formulations can be analyzed.

## 5 Conclusion

In this observational trial, we found non-therapeutic curcumin plasma levels in patients and healthy participants using commonly available and homemade formulations of curcumin. Our results confirm that curcumin is rapidly and completely metabolized after oral intake. Several studies make claims of high curcumin concentrations, but they actually report total curcumin levels, which mainly consists of inactive conjugates. This is a misleading claim, and therefore, we emphasize the need to approach the claimed beneficial health effects of curcumin supplement use in daily life with caution. We believe that health care practitioners have an important role in providing patients with accurate information regarding the use of curcumin for health benefits.

## Data availability statement

The raw data supporting the conclusions of this article will be made available by the authors, without undue reservation.

## Ethics statement

The studies involving humans were approved by Ethics Committee Amsterdam UMC, location Academic Medical Center. The studies were conducted in accordance with the local legislation and institutional requirements. The participants provided their written informed consent to participate in this study.

## Author contributions

MK: Conceptualization, Data curation, Formal analysis, Investigation, Methodology, Validation, Visualization, Writing – original draft, Writing – review & editing. JB: Conceptualization, Investigation, Methodology, Visualization, Writing – original draft. SM: Investigation, Writing – review & editing. ES: Resources, Supervision, Writing – review & editing. OT: Data curation, Supervision, Validation, Writing – review & editing. HL: Resources, Supervision, Writing – review & editing. EK: Conceptualization, Data curation, Methodology, Project administration, Resources, Supervision, Writing – review & editing.

## References

[1] Chen M du ZY Zheng X Li DL Zhou RP Zhang K. Use of curcumin in diagnosis, prevention, and treatment of Alzheimer's disease. Neural Regen Res. (2018) 13:742–52. doi: 10.4103/1673-5374.230303, PMID: 29722330 PMC5950688

[2] Wojcik M Krawczyk M Wojcik P Cypryk K Wozniak LA. Molecular mechanisms underlying curcumin-mediated therapeutic effects in type 2 diabetes and Cancer. Oxidative Med Cell Longev. (2018) 2018:9698258. doi: 10.1155/2018/9698258 PMC5884026 29743988

[3] Rahmani AH Alsahli MA Aly SM Khan MA Aldebasi YH. Role of curcumin in disease prevention and treatment. Adv Biomed Res. (2018) 7:38. doi: 10.4103/abr.abr_147_16 29629341 PMC5852989

[4] Li L Braiteh FS Kurzrock R. Liposome-encapsulated curcumin: in vitro and in vivo effects on proliferation, apoptosis, signaling, and angiogenesis. Cancer. (2005) 104:1322–31. doi: 10.1002/cncr.21300, PMID: 16092118

[5] Mach CM Mathew L Mosley SA Kurzrock R Smith JA. Determination of minimum effective dose and optimal dosing schedule for liposomal curcumin in a xenograft human pancreatic cancer model. Anticancer Res. (2009) 29:1895–9. PMID: 19528445

[6] Ringman JM Frautschy SA Teng E Begum AN Bardens J Beigi M. Oral curcumin for Alzheimer's disease: tolerability and efficacy in a 24-week randomized, double blind, placebo-controlled study. Alzheimers Res Ther. (2012) 4:43. doi: 10.1186/alzrt146, PMID: 23107780 PMC3580400

[7] Hurlstone DP Karajeh M Sanders DS Drew SK Cross SS. Rectal aberrant crypt foci identified using high-magnification-chromoscopic colonoscopy: biomarkers for flat and depressed neoplasia. Am J Gastroenterol. (2005) 100:1283–9. doi: 10.1111/j.1572-0241.2005.40891.x, PMID: 15929758

[8] Dhillon N Aggarwal BB Newman RA Wolff RA Kunnumakkara AB Abbruzzese JL. Phase II trial of curcumin in patients with advanced pancreatic cancer. Clin Cancer Res. (2008) 14:4491–9. doi: 10.1158/1078-0432.CCR-08-0024, PMID: 18628464

[9] Lao CD Ruffin MT IV Normolle D Heath DD Murray SI Bailey JM. Dose escalation of a curcuminoid formulation. BMC Complement Altern Med. (2006) 6:10. doi: 10.1186/1472-6882-6-10, PMID: 16545122 PMC1434783

[10] Metzler M Pfeiffer E Schulz SI Dempe JS. Curcumin uptake and metabolism. Biofactors. (2013) 39:14–20. doi: 10.1002/biof.1042 22996406

[11] Wong KE Ngai SC Chan KG Lee LH Goh BH Chuah LH. Curcumin Nanoformulations for colorectal Cancer: a review. Front Pharmacol. (2019) 10:152. doi: 10.3389/fphar.2019.00152, PMID: 30890933 PMC6412150

[12] Shoba G Joy D Joseph T Majeed M Rajendran R Srinivas P. Influence of piperine on the pharmacokinetics of curcumin in animals and human volunteers. Planta Med. (1998) 64:353–6. doi: 10.1055/s-2006-957450, PMID: 9619120

[13] Berginc K Trontelj J Basnet NS Kristl A. Physiological barriers to the oral delivery of curcumin. Pharmazie. (2012) 67:518–24. PMID: 22822540

[14] Patil VM Das S Balasubramanian K. Quantum chemical and docking insights into bioavailability enhancement of curcumin by Piperine in pepper. J Phys Chem A. (2016) 120:3643–53. doi: 10.1021/acs.jpca.6b01434 27111639

[15] Mukkavilli R Gundala SR Yang C Jadhav GR Vangala S Reid MD. Noscapine recirculates enterohepatically and induces self-clearance. Eur J Pharm Sci. (2015) 77:90–9. doi: 10.1016/j.ejps.2015.05.026, PMID: 26026989 PMC4516653

[16] Sasaki H Sunagawa Y Takahashi K Imaizumi A Fukuda H Hashimoto T. Innovative preparation of curcumin for improved oral bioavailability. Biol Pharm Bull. (2011) 34:660–5. doi: 10.1248/bpb.34.660, PMID: 21532153

[17] Morimoto T Sunagawa Y Katanasaka Y Hirano S Namiki M Watanabe Y. Drinkable preparation of Theracurmin exhibits high absorption efficiency-a single-dose, double-blind, 4-way crossover study. Biol Pharm Bull. (2013) 36:1708–14. doi: 10.1248/bpb.b13-00150, PMID: 24189415

[18] Kanai M Otsuka Y Otsuka K Sato M Nishimura T Mori Y. A phase I study investigating the safety and pharmacokinetics of highly bioavailable curcumin (Theracurmin) in cancer patients. Cancer Chemother Pharmacol. (2013) 71:1521–30. doi: 10.1007/s00280-013-2151-8, PMID: 23543271

[19] Awolade P Cele N Kerru N Gummidi L Oluwakemi E Singh P. Therapeutic significance of β-glucuronidase activity and its inhibitors: a review. Eur J Med Chem. (2020) 187:111921. doi: 10.1016/j.ejmech.2019.111921, PMID: 31835168 PMC7111419

[20] Ho KJ. Human beta-glucuronidase.Studies on the effects of pH and bile acids in regard to its role in the pathogenesis of cholelithiasis. Biochim Biophys Acta. (1985) 827:197–206. doi: 10.1016/0167-4838(85)90203-1, PMID: 3970937

[21] Kroon M van Laarhoven HWM Swart EL Kemper EM van Tellingen O. A validated HPLC-MS/MS method for simultaneously analyzing curcumin, demethoxycurcumin, bisdemethoxycurcumin, tetrahydrocurcumin and piperine in human plasma, urine or feces. Heliyon. (2023) 9:e15540. doi: 10.1016/j.heliyon.2023.e15540, PMID: 37131436 PMC10149208

[22] Nelson KM Dahlin JL Bisson J Graham J Pauli GF Walters MA. The essential medicinal chemistry of curcumin. J Med Chem. (2017) 60:1620–37. doi: 10.1021/acs.jmedchem.6b00975, PMID: 28074653 PMC5346970

[23] Yue GG Chan BC Hon PM Lee MY Fung KP Leung PC. Evaluation of in vitro anti-proliferative and immunomodulatory activities of compounds isolated from *Curcuma longa*. Food Chem Toxicol. (2010) 48:2011–20. doi: 10.1016/j.fct.2010.04.039, PMID: 20438793 PMC2910176

[24] Maiti P Manna J Thammathong J Evans B Dubey KD Banerjee S. Tetrahydrocurcumin has similar anti-amyloid properties as curcumin: in Vitro comparative structure-activity studies. Antioxidants. (2021) 10:1592. doi: 10.3390/antiox10101592 34679727 PMC8533373

[25] Li J Wang S Zhang S Cheng D Yang X Wang Y. Curcumin slows the progression of Alzheimer's disease by modulating mitochondrial stress responses via JMJD3-H3K27me3-BDNF axis. Am J Transl Res. (2021) 13:13380–93. 35035682 PMC8748089

[26] Omeroglu Ulu Z Degirmenci NS Bolat ZB Sahin F. Synergistic anti-cancer effect of sodium pentaborate pentahydrate, curcumin and piperine on hepatocellular carcinoma cells. Sci Rep. (2023) 13:14404. doi: 10.1038/s41598-023-40809-y 37658091 PMC10474293

[27] Gota VS Maru GB Soni TG Gandhi TR Kochar N Agarwal MG. Safety and pharmacokinetics of a solid lipid curcumin particle formulation in osteosarcoma patients and healthy volunteers. J Agric Food Chem. (2010) 58:2095–9. doi: 10.1021/jf9024807, PMID: 20092313

[28] Schiborr C Kocher A Behnam D Jandasek J Toelstede S Frank J. The oral bioavailability of curcumin from micronized powder and liquid micelles is significantly in-creased in healthy humans and differs between sexes. Mol Nutr Food Res. (2014) 58:516–27. doi: 10.1002/mnfr.201300724, PMID: 24402825

[29] Kunihiro AG Luis PB Brickey JA Frye JB Chow HHS Schneider C. Beta-Glucuronidase catalyzes Deconjugation and activation of curcumin-glucuronide in bone. J Nat Prod. (2019) 82:500–9. doi: 10.1021/acs.jnatprod.8b00873, PMID: 30794412 PMC6528680

[30] Ireson C Orr S Jones DJ Verschoyle R Lim CK Luo JL. Characterization of metabolites of the chemopreventive agent curcumin in human and rat hepatocytes and in the rat in vivo, and evaluation of their ability to inhibit phorbol ester-induced prostaglandin E2 production. Cancer Res. (2001) 61:1058–64. PMID: 11221833

[31] Ozawa H Imaizumi A Sumi Y Hashimoto T Kanai M Makino Y. Curcumin beta-D-glucuronide plays an important role to keep high levels of free-form curcumin in the blood. Biol Pharm Bull. (2017) 40:1515–24. doi: 10.1248/bpb.b17-00339, PMID: 28867734

[32] Kanai M Imaizumi A Otsuka Y Sasaki H Hashiguchi M Tsujiko K. Dose-escalation and pharmacokinetic study of nanoparticle curcumin, a potential anticancer agent with improved bioavailability, in healthy human volunteers. Cancer Chemother Pharmacol. (2012) 69:65–70. doi: 10.1007/s00280-011-1673-1, PMID: 21603867

[33] Kocher A Schiborr C Behnam D Frank J. The oral biovailability of curcuminoids in healthy humans is markedly enhanced by micellar solubilisation but not further improved by simultaneous ingestion of sesamin, ferulic acid, naringenin and xanthohumol. J Funct Foods. (2015) 14:183–91. doi: 10.1016/j.jff.2015.01.045

[34] Wang YJ Pan MH Cheng AL Lin LI Ho YS Hsieh CY. Stability of curcumin in buffer solutions and characterization of its degradation products. J Pharm Biomed Anal. (1997) 15:1867–76. doi: 10.1016/S0731-7085(96)02024-9, PMID: 9278892

[35] Volak LP Ghirmai S Cashman JR Court MH. Curcuminoids inhibit multiple human cytochromes P450, UDP-glucuronosyltransferase, and sulfotransferase enzymes, whereas piperine is a relatively selective CYP3A4 inhibitor. Drug Metab Dispos. (2008) 36:1594–605. doi: 10.1124/dmd.108.020552, PMID: 18480186 PMC2574793

[36] Vecchi Brumatti L Marcuzzi A Tricarico P Zanin V Girardelli M Bianco A. Curcumin and inflammatory bowel disease: potential and limits of innovative treatments. Molecules. (2014) 19:21127–53. doi: 10.3390/molecules191221127, PMID: 25521115 PMC6271352

